# Colistin Resistance: From Laboratory Research to Modern Clinical Management

**DOI:** 10.3390/antibiotics15030259

**Published:** 2026-03-02

**Authors:** Hakan Erdem, Gulden Yilmaz-Tehli

**Affiliations:** Department of Infectious Diseases and Clinical Microbiology, Gülhane School of Medicine, Health Sciences University, Ankara 06010, Türkiye; drguldeny@yahoo.com.tr

**Keywords:** colistin, polymyxins, antimicrobial resistance, multidrug-resistant Gram-negative bacteria, *Klebsiella pneumoniae*

## Abstract

**Background/Objectives:** Colistin, a polymyxin antibiotic introduced in the mid-20th century, has regained clinical importance as a last-resort agent for the treatment of infections caused by multidrug-resistant (MDR) Gram-negative bacteria. The global dissemination of carbapenem-resistant pathogens has intensified colistin use, leading to a concerning rise in resistance. This review aims to provide a comprehensive and up-to-date synthesis of colistin’s pharmacological characteristics, resistance mechanisms, epidemiology, and current and emerging therapeutic strategies. **Methods:** A narrative review of the literature was conducted, encompassing studies on the chemistry, mechanism of action, pharmacodynamics, clinical use, dosing, and resistance to colistin. Data on chromosomal and plasmid-mediated resistance mechanisms, detection methodologies, epidemiological trends, and clinical outcomes were examined. In addition, evidence on colistin-based treatment strategies and novel non-antibiotic approaches was analyzed. **Results:** Colistin remains active against many MDR Gram-negative pathogens, including *Klebsiella pneumoniae*, *Pseudomonas aeruginosa*, and *Acinetobacter baumannii*; however, resistance is increasingly reported worldwide. Both chromosomally mediated modifications of lipid A and plasmid-mediated mcr genes contribute to resistance, with heteroresistance posing diagnostic and therapeutic challenges. Carbapenem resistance has emerged as a major driver of colistin use and subsequent resistance selection. Combination therapies, inhaled formulations, and guideline-directed use may improve outcomes, while emerging alternatives such as antimicrobial peptides, bacteriophages, nanoparticles, photodynamic therapy, and CRISPR-based technologies show promise. **Conclusions:** The escalating prevalence of colistin resistance threatens the effectiveness of this critical last-line antibiotic. Optimized use, robust resistance surveillance, accurate detection methods, and the development of innovative therapeutic strategies are essential to preserve colistin’s clinical utility and address the growing burden of MDR Gram-negative infections.

## 1. Introduction

Antibiotics have been a cornerstone of modern medicine, but their effectiveness is increasingly threatened by antimicrobial resistance (AMR), now recognized as a major global public health challenge [1,2]. AMR increases healthcare costs, prolongs hospital stays, and raises mortality. Projections have warned that it can cause up to 10 million deaths annually by 2050 [3]. Although AMR occurs naturally, its acceleration is driven by inappropriate antibiotic use across human, veterinary, and agricultural sectors. Environmental pathways, including food and aerosols, further facilitate spread, reinforcing the importance of a One Health approach linking human, animal, and environmental health [4,5].

The emergence of resistance to last-resort antibiotics for multidrug-resistant Gram-negative infections is a growing concern. Colistin, a polymyxin reintroduced due to the scarcity of alternative therapies, has become a vital treatment option. However, the increasing detection of colistin-resistant organisms threatens its clinical utility and signals a critical juncture in the AMR crisis. This review not only describes colistin resistance through its molecular mechanisms but also offers a integrated perspective by addressing the issue in the context of clinical use, diagnostics, treatment strategies, and patient outcomes. It focuses on practical decision-making processes, including pharmacokinetic properties, current guideline recommendations, and antibiotic combination therapies. In addition, by discussing innovative non-antibiotic approaches and evaluating potential therapeutic options for the post-colistin era, the article provides a timely and forward-looking perspective.

## 2. Overview of Colistin

### 2.1. Polymyxins

Polymyxins are cyclic cationic polypeptide detergents and consist of five groups (A, B, C, D, and E). Of clinical significance, polymyxin B is derived from *Bacillus polymyxa*, whereas colistin (polymyxin E) is obtained from *Bacillus colistinus*. Colistin was first isolated in 1947 and began to be widely used in intravenous formulations in Japan and Europe. In 1959, it was approved by the United States Food and Drug Administration for the treatment of infections due to multidrug resistant Gram-negative bacteria (MDR-GNB) including those caused by *P. aeruginosa*, *Klebsiella pneumoniae*, and *A. baumannii* [6]. In the following years, its clinical use was largely restricted due to concerns regarding significant neurotoxicity and nephrotoxicity. However, with the increasing incidence of infections caused by MDR bacteria [7], colistin has gradually been reintroduced into clinical practice [6,8]. Given its narrow therapeutic window, the judicious use of colistin is essential and requires a comprehensive understanding of its pharmacokinetics, pharmacodynamics, mechanisms of action, and resistance pathways. Accordingly, this paper focuses on optimizing the clinical use of colistin.

### 2.2. Human Use

Colistin is considered one of the last-resort antibiotics against MDR-GNB, including carbapenemase-producing Enterobacteriaceae, *Pseudomonas* spp., and *Acinetobacter* spp. today. It is particularly used in critical clinical patients with bacteremia/sepsis and ventilator-associated pneumonia in intensive care units (ICUs). In addition, colistin is regarded as an alternative treatment option for various clinical conditions, including urinary tract infections, osteomyelitis, joint infections, meningitis, pneumonia, gastrointestinal tract infections, soft tissue infections, as well as eye and ear infections [6,8].

### 2.3. Veterinary Use

The drug has also been widely used in veterinary medicine. It has frequently been employed for the prophylaxis and treatment of intestinal infections in pigs, calves, and poultry, as well as to promote growth in pigs and fish [9,10,11]. A study in Vietnam showed mcr-1 colistin resistance was highly prevalent in chickens exposed to colistin and was also detected in farmers who had contact with them, indicating direct zoonotic transmission of resistant *E. coli* [12]. Accordingly, research from Portugal identified colistin-resistant *E. coli* carrying the mcr-1 gene in both companion animals and the household humans; some isolates were genetically indistinguishable, implying direct sharing of resistance between animals and people [13]. Thus, the emergence of antimicrobial-resistant bacteria resulting from the excessive and inappropriate use of colistin has led to restrictions on its veterinary use in some countries, such as Canada. Moreover, the Chinese government has banned the use of colistin in animal feed [10,11].

### 2.4. Mechanism of Activity

**Figure 1:**
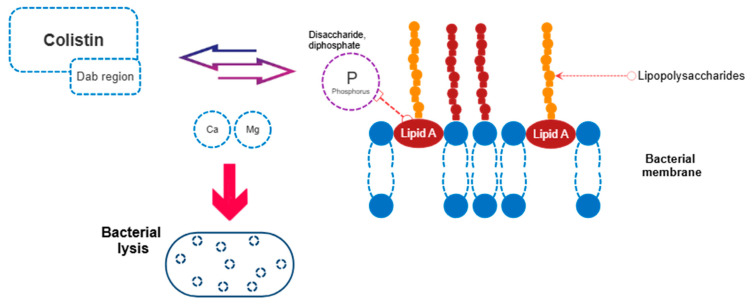
Mechanism of action for colistin. **Dab:** α,γ-Diaminobutyric Acid.

Colistin exerts its antibacterial activity through a couple of mechanisms. The classical mechanism involves an electrostatic interaction between the α,γ-diaminobutyric acid component of colistin and the phosphate groups of lipid A within the lipopolysaccharide (LPS) layer of the outer cell membrane. This interaction leads to displacement of divalent cations (Ca^2+^ and Mg^2+^), resulting in destabilization of the phospholipid bilayer, loss of membrane integrity, increased permeability, leakage of intracellular contents, and ultimately cell lysis [6] (Figure 1). In addition, colistin can induce cell lysis through alternative pathways. These include binding to anionic phospholipid vesicles and disrupting intracellular osmotic balance via phospholipid exchange between vesicles; inducing oxidative stress through the generation of superoxide, peroxide, and hydroxyl radicals; or inhibiting key respiratory enzymes in the inner membrane, particularly type II NADH oxidoreductase, thereby impairing the bacterial electron transport system and leading to cell death. Furthermore, by interacting with the lipid A component of the LPS layer, colistin also exhibits anti-endotoxin activity [6,9,10].

### 2.5. In Vitro Pharmacodynamics

Polymyxins in vitro exhibit rapid concentration-dependent bactericidal activity. A post-antibiotic effect has been observed against *P. aeruginosa*, whereas such an effect is not evident for *A. baumannii* or *K. pneumoniae* [14,15]. The area under the concentration, time curve to minimum inhibitory concentration ratio (AUC/MIC) appears to be the pharmacodynamic parameter most closely associated with bactericidal activity for polymyxins. Based on a target mean steady-state concentration of 2 µg/mL and 50% protein binding, the reported mean AUC/MIC ratio necessary for bactericidal activity is almost 24 [6].

### 2.6. Spectrum of Activity

Polymyxins are active against a broad range of aerobic Gram-negative bacilli, with the exception of intrinsically resistant *Proteus* species. However, they exhibit limited activity against *Providencia*, *Burkholderia*, *Serratia*, *Moraxella*, *Helicobacter*, *Campylobacter*, *Vibrio*, *Brucella*, *Aeromonas*, *Morganella*, and *Edwardsiella* species. The antibacterial activity of polymyxins is reduced in the presence of divalent cations such as calcium and magnesium [6,8,9].

### 2.7. Administration of Colistin

Colistin is available in two forms: colistin sulfate and colistimethate sodium (CMS). CMS is a sulfomethylated derivative of colistin and is an inactive prodrug that must undergo hydrolysis to be converted into its active form to exert antibacterial activity. Colistin sulfate is used via topical and oral routes, whereas CMS is administered intramuscularly, intravenously, and by inhalation. More rarely, in appropriate clinical situations, both colistin and polymyxin B may be administered intrathecally or intraventricularly. In the United States, both CMS and polymyxin formulations are available, while in some other countries only one of these forms may be accessible [6].

### 2.8. Dosing of Colistin

In vials manufactured in the United States, each formulation contains 360 mg of CMS, corresponding to 150 mg of colistin base activity (CBA), which is equivalent to 4.5 million international units (MIU). In the literature, 1 MIU is generally accepted to be equivalent to approximately 33 mg of CBA [6]. Colistin dosing should be guided by the recommendations outlined in the international consensus guidelines for the optimal use of polymyxins, which are based on current pharmacokinetic and pharmacodynamic evidence [16]. These guidelines propose a target plasma concentration of 2 µg/mL, taking both efficacy and safety into consideration; however, the optimality of this target concentration remains uncertain. Importantly, toxicodynamic data indicate that mean steady-state concentrations exceeding 2 µg/mL are associated with an increased frequency and severity of nephrotoxicity. Based on these findings, a loading dose of 300 mg of CBA (9 MIU) of CMS administered by infusion over 0.5–1 h is recommended, followed by a maintenance regimen of 300–360 mg of CBA (9–10.9 MIU) per day, divided into two doses and infused over 0.5–1 h at 12 h intervals.

### 2.9. Dose Adjustments

CMS dosing must be adjusted in patients with renal impairment based on creatinine clearance. For patients undergoing intermittent hemodialysis, a dose of 130 mg CBA/day (3.9 MIU/day) is recommended on non-dialysis days, with an additional supplemental dose of 40 mg CBA (1.2 MIU) on dialysis days, resulting in a total daily dose of 170 mg CBA (5.2 MIU). In patients receiving continuous renal replacement therapy, a total daily dose of 440 mg CBA (13.3 MIU), administered in two divided doses, is recommended [6,16].

## 3. Resistance Mechanisms

MDR-GNB, which can disseminate extensively via mobile genetic elements such as plasmids, constitute a substantial proportion of the AMR burden [1]. GNB possess the capacity to develop resistance through multiple mechanisms, including enzymatic modification of antibiotics (e.g., β-lactamase production), reduced intracellular antibiotic uptake due to porin loss, alterations at the antibiotic target site (e.g., ribosomal modifications), and increased efflux from the cell through overexpression of efflux pumps. These resistance mechanisms arise through mutations in the bacterial chromosome and, more commonly, through the acquisition of novel resistance determinants mediated by plasmids. Importantly, mobile genetic elements can harbor resistance genes conferring resistance to multiple antibiotic classes simultaneously. Colistin resistance, in particular, is a major concern because the drug is a last-resort antibiotic used to treat life-threatening infections caused by MDR-GNB bacteria. The emergence of transferable genes enables rapid spread of colistin resistance across humans, animals, and the environment, severely limiting treatment options and increasing mortality [17,18]. Colistin resistance is broadly categorized into two main mechanisms: chromosomally mediated and plasmid-mediated resistance.

### 3.1. Chromosomally Mediated Colistin Resistance

One of the principal mechanisms underlying this type of resistance is modification of the lipopolysaccharide (LPS) layer through cation substitution. As a result of this modification, phosphoethanolamine (PEtN) and 4-amino-4-deoxy-L-arabinose (L-Ara4N) are added to the lipid A moiety of LPS, leading to a reduction in the net negative charge of the outer membrane. This decrease in negative charge diminishes electrostatic interactions, thereby reducing colistin affinity and resulting in colistin resistance [6,10]. In GNB, colistin resistance is regulated by the PmrA–PmrB (PmrAB) and PhoP–PhoQ (PhoPQ) two-component systems (operons or regulatory pathways). While the PmrAB system primarily controls lipid A modification, the PhoPQ system is activated under conditions such as low magnesium or calcium concentrations or acidic pH, and promotes lipid A modification through two distinct pathways, thereby conferring bacterial protection [19]. Chromosomal mutations within these regulatory systems have been associated with colistin resistance in a wide range of GNB, including *K. pneumoniae*, *P. aeruginosa*, *Salmonella* spp., *A. baumannii*, *E. coli*, and *Enterobacter* spp. [19]. In addition, colistin resistance may also arise through the activity of various efflux pumps. For example, upon exposure to antibiotics, *P. aeruginosa* can acquire resistance to colistin by upregulating the expression of the MexAB–OprM efflux pump [6,9,10]. Since the interaction between polymyxins and lipopolysaccharide (LPS) is essential for the bactericidal activity of polymyxins, complete loss of LPS can also result in colistin resistance in *A. baumannii* [9,10].

### 3.2. Plasmid Mediated Resistance

Before 2015, colistin resistance in GNB was thought to be primarily driven by chromosomal modifications, particularly those affecting genes and operons involved in lipid A biosynthesis. However, in 2016, the first plasmid-mediated colistin resistance gene, mcr-1, was identified in *E. coli* isolates obtained from pigs in China [11]. Mobile colistin resistance (mcr) genes are plasmid-borne determinants that confer colistin resistance on a broader scale through horizontal gene transfer. The mcr gene, a member of the PEtN transferase family, catalyzes the addition of PEtN to the lipid A moiety of the LPS layer, thereby reducing colistin binding affinity to the bacterial membrane and diminishing its antimicrobial activity [10]. Horizontal dissemination of the plasmid-borne mcr-1 gene has facilitated widespread colistin resistance among diverse GNB and has even reached the human food chain, raising significant public health concerns. According to a study by Poirel et al., the presence of mcr-1 results in a 4- to 8-fold increase in colistin MICs [17]. Notably, a high prevalence of the mcr-1 gene has been detected in *E. coli* isolates recovered from pigs and poultry, underscoring the extensive use of colistin in livestock farming for the treatment of GNB infections [20]. Following its initial identification in China, mcr-1 has been reported worldwide in various GNB species. Multiple plasmid incompatibility types have been associated with mcr-1 dissemination, including IncX4, IncHI1, IncHI2, IncP, IncI2, and IncY, with IncI2 and IncX4 being the most frequently identified [10]. Recent studies have revealed that some mcr-carrying plasmids also harbor carbapenemase resistance genes, such as NDM-1, NDM-5, KPC-2, and KPC-3, along with colistin resistance determinants [10,21,22]. The co-localization of mcr genes with other antibiotic resistance genes, particularly the carbapenemases, on the same conjugative plasmid promotes co-selection and dissemination of these MDR isolates, posing a substantial challenge to current therapeutic options [10]. More than 25 variants of the mcr-1 gene have been identified, each differing by one or two amino acids. To date, ten distinct mcr genes (mcr-1 to mcr-10) have been described [19]. The resistance mechanism, PEtN transfer to lipid A, is conserved across all allelic variants, although sequence variability suggests diverse genetic origins. Approximately 91% of all mcr-positive isolates are *E. coli*, followed by *Salmonella enterica* (7%) and *K. pneumoniae* (2%) [23]. The details of mcr genes associated with plasmid-mediated colistin resistance are presented in Table 1 [24].

### 3.3. Effects of Cell Metabolism on Colistin Resistance

Recent studies demonstrate that the metabolic state of the cell markedly influences antibiotic efficacy as well as the development of antimicrobial resistance. This interaction is bidirectional: bacterial metabolism shapes the response to antibiotic treatment, while antibiotics may exert variable effects depending on the metabolic environment of both the bacterium and the host [25]. Enhancement of cellular metabolism through the exogenous supplementation of metabolites and/or activation of specific metabolic pathways can resensitize resistant bacteria to antibiotic therapy. Moreover, increasing metabolic activity within biofilms is predicted to potentiate the bactericidal effects of antibiotics. Collectively, these findings highlight metabolism-targeted strategies as promising and innovative approaches in the fight against antimicrobial resistance [26,27,28].

Porins facilitate the passage of hydrophilic nutrients and antibiotics across the outer membrane and also cause proton leakage from the periplasm, thereby altering membrane conductance and permeability. Porin permeability increases when periplasmic H^+^ levels are low during starvation and decreases with periplasmic acidification following nutrient uptake. In glucose-containing environments, high metabolic activity activates voltage-gated potassium channels in the inner membrane, leading to increased periplasmic K^+^ levels and enhanced porin permeability to help dissipate reactive oxygen species. This metabolic control of porin permeability suggests that mutations in core metabolic genes may influence antibiotic resistance and indicates that potassium channels may be important therapeutic targets. Accordingly, a study by Cano-Muñiz et al. demonstrated that porin permeability in *E. coli* is regulated by changes in periplasmic H^+^ and K^+^ concentrations associated with metabolic states, and emphasized that voltage-gated K^+^ channels could serve as therapeutic targets to enhance antibiotic efficacy [29].

To assess the contribution of bacterial growth state and metabolic activity to Polymyxin B–mediated killing, equal numbers of *E. coli* cells in the logarithmic (metabolically active) and stationary (metabolically reduced) phases were treated with Polymyxin B at a defined concentration (4 µg ml^−1^). Subpopulations from each growth phase were cultured in the presence of glucose to enhance metabolic activity. Under glucose-supplemented conditions, Polymyxin B exhibited potent bactericidal activity against stationary-phase cells. In contrast, stationary-phase *E. coli* exposed to Polymyxin B in the absence of glucose showed no measurable loss of viability. Consequently, these results demonstrate that active metabolism is a critical determinant of the bactericidal efficacy of Polymyxin B [30]. In a recent study, the absence of three membrane transport proteins in *E. coli* like ClcB (a chloride channel protein), PtsI (a component of the phosphotransferase sugar transport system), and YcaM (a GABA antiporter) was shown to reduce susceptibility to colistin. This study provided the first evidence that disruptions in these transporter proteins modulate colistin susceptibility [31].

### 3.4. Collateral Effects of Colistin Resistance

Molecular analyses have demonstrated that colistin resistance is frequently associated with loss or modification of LPS. While these mechanisms confer resistance, studies, particularly in murine models, have shown that they may also impose other alterations on bacteria. These changes include reduced virulence, increased susceptibility to lysozyme and lactoferrin, slower growth rates, decreased production of inflammatory cytokines, reduced biofilm formation, and lower mortality rates [32].

## 4. Detection and Classification of Colistin Resistance

### 4.1. Methods for Detecting Colistin Resistance

Both phenotypic and molecular methods are employed for the detection of colistin resistance. Phenotypic methods include selective agar media, broth microdilution, automated susceptibility systems, MALDI-TOF–based assays, rapid colorimetric tests, and enzyme inhibition assays; these methods vary in speed and practicality but often provide limited insight into resistance mechanisms [10]. Molecular methods, such as whole genome sequencing, PCR-based assays, LAMP, and other genotyping techniques, offer faster and more precise identification of resistance genes, particularly plasmid-mediated mcr variants, though they are more costly and require specialized expertise [33,34].

### 4.2. Colistin Susceptibility Testing

The rapid and reliable identification of resistant bacterial isolates recovered in clinical settings is of critical importance [10]. For the determination of colistin minimum inhibitory concentrations (MICs) in pure bacterial isolates, both the European Committee on Antimicrobial Susceptibility Testing (EUCAST) and the Clinical and Laboratory Standards Institute (CLSI) recommend the broth microdilution method as the gold standard [6]. However, discrepancies exist between CLSI and EUCAST with respect to susceptibility breakpoints. CLSI does not define susceptibility breakpoints for colistin or polymyxin B; instead, it categorizes *P. aeruginosa*, *A. baumannii*, and members of the Enterobacterales family with MIC values ≤ 2 µg/mL as intermediate [6]. In contrast, EUCAST provides breakpoints only for colistin, as polymyxin B is not available in Europe, and defines epidemiological cutoff values of 2 µg/mL for *Acinetobacter* spp. and Enterobacterales, and 4 µg/mL for *Pseudomonas* spp. [35].

### 4.3. Antimicrobial Resistance Phenotypes

Standardized definitions for AMR were established in 2012 [36]. Resistance to at least one agent in three or more antimicrobial classes is defined as multidrug resistance; resistance to all but one or two antimicrobial classes is defined as extensively drug-resistant (XDR); and resistance to all available antimicrobial classes is defined as pandrug-resistant (PDR) [36]. In more recent years, the term difficult-to-treat resistance has been introduced and is defined as resistance to all first-line agents, including carbapenems, β-lactam/β-lactamase inhibitor combinations (excluding newer agents), and fluoroquinolones [37].

## 5. Epidemiology of Colistin Resistance

### 5.1. Emerging Colistin Resistance

The first report of colistin resistance dates back to 1947 [38], yet such resistance was reported only sporadically until the early 2000s. The situation has further worsened with the rapid spread of this resistance, particularly over the past decade [39]. A modest increase was observed between 2009 and 2015; however, since 2015, the prevalence of colistin resistance has risen markedly [6]. Furthermore, although newly developed antibiotics such as plazomicin, siderophore-based agents, eravacycline, and meropenem/vaborbactam have strengthened the fight against colistin-resistant *K. pneumoniae*, the emergence of strains already resistant to these novel drugs like ceftazidime/avibactam, meropenem/vaborbactam, and eravacycline is highly concerning [40,41].

Colistin-resistant GNB can cause nosocomial outbreaks, too. In 2016, a small-scale outbreak due to colistin-resistant *K. pneumoniae* at a referral hospital in Türkiye resulted in the deaths of five infected patients [42]. During the COVID-19 pandemic, extensive use of broad-spectrum antibiotics, including colistin, particularly in critically ill patients, along with prolonged hospitalization and increased mechanical ventilation, was associated with increased multidrug-resistant infections [43]. Despite this trend, polymyxins remain still active against many MDR-GNB, including *P. aeruginosa*, *A. baumannii*, and CRE, with MIC_90_ values ≤ 2 µg/mL for the majority of isolates. Nevertheless, an increasing number of studies have documented polymyxin resistance rates exceeding 10%, particularly in regions where CRE is endemic. In some reports, polymyxin resistance has been identified in more than half of the isolates [44,45].

It is well recognized that colistin resistance is not uncommon even in bacteremias occurring in immunocompromised patients with febrile neutropenia that are presumed to originate from the patients’ endogenous flora. A reasonable explanation appears to be the frequent exposure of these patients to healthcare settings leading to leakage of the colistin resistant strains to the community [46]. Thus, caution is warranted regarding the potential dissemination of colistin resistance into community-acquired pathogens, mirroring the historical transition observed in *Staphylococcus aureus* isolates, which have evolved to cause community-onset MRSA outbreaks across Europe today [47]. Table 2 presents the meta-analyses on colistin resistance [48,49,50,51,52,53,54,55].

### 5.2. Heteroresistance to Colistin and Its Clinical Implications

Heteroresistance is commonly observed among polymyxin-susceptible strains. Colistin heteroresistance refers to the presence of subpopulations that can grow at colistin concentrations > 2 µg/mL within isolates exhibiting a colistin MIC < 2 µg/mL, and is thought to arise following colistin exposure [56,57]. Notably, cross-resistance between colistin and polymyxin B has also been reported [6]. In a study conducted by Band et al. across eight states in the United States, colistin heteroresistance was identified in 10.1% of 408 isolates collected for CRE surveillance, and was noted to be more prevalent than conventional homogeneous resistance (7.1%) [58]. The studies on carbapenem-resistant *A. baumannii* and Carbapenem-resistant Enterobacterales infections demonstrated that colistin heteroresistance is frequently undetected by routine susceptibility testing, resulting in misclassification as colistin-susceptible. During therapy, heteroresistant strains can rapidly evolve into fully resistant phenotypes, a process significantly associated with treatment failure and increased 14-day mortality, particularly in bacteremic patients [58,59]. These findings highlight colistin heteroresistance as a clinically important determinant of poor outcomes and support the use of alternative or combination therapies rather than colistin monotherapy. Consequently, colistin, which was previously classified under the highly important category in the World Health Organization list of antimicrobial agents of importance to human health (critically important/highly important/important), has now been reclassified into the critically important category due to increasing resistance rates [60].

### 5.3. Carbapenem Resistance as a Driver of Colistin Resistance

Since the turn of the 21st century, carbapenem resistance among key Gram-negative pathogens including *Acinetobacter* species, *P. aeruginosa*, and Enterobacterales has emerged as a major global clinical challenge [61,62]. In one study, the duration of carbapenem therapy was identified as an independent risk factor for colistin-resistant *A. baumannii* infection [63]. The coexistence of carbapenem and colistin resistance imposes a serious clinical dilemma. In another study, 24.2% of carbapenem-resistant *K. pneumoniae* bloodstream isolates and 4.8% of *P. aeruginosa* isolates were found to be resistant to colistin, further underscoring this concern [64]. Collectively, coexistent carbapenem and colistin resistance denotes resistance to two last-line antimicrobial classes, resulting in severely limited treatment options and posing a substantial clinical and public health threat.

### 5.4. Colistin Resistance Among K. pneumoniae

In a meta-analysis evaluating colistin resistance in *K. pneumoniae* isolates obtained from bloodstream infections, the overall prevalence of colistin resistance was found to be 3.1% (95% CI: 1.5–4.7%). Another meta-analysis of bacteremic patients identified *K. pneumoniae* as having the highest rate of colistin resistance, which increased from 2.9% prior to 2020 to 12.9% after 2020. Notably, higher resistance rates were observed in isolates recovered from patients hospitalized in ICUs compared with those from non-ICU settings (ICU: 11.5% vs. non-ICU: 3.03%) [65]. In a review evaluating 28 studies conducted in Türkiye, the prevalence of colistin resistance among *K. pneumoniae* isolates was calculated to be 13.5% by broth microdilution method, and a significant increase in resistance rates over time was emphasized [50]. Overall, it appears that colistin resistance in *K. pneumoniae* is rapidly escalating and poses a growing threat to the effective management of severe infections, particularly in high-risk hospital settings (see Table 2).

### 5.5. Colistin Resistance Among P. aeruginosa

Although the level of colistin resistance in *P. aeruginosa* is lower compared with other GNB, resistance continues to increase steadily. In a meta-analysis evaluating colistin resistance globally in clinical *P. aeruginosa* isolates between 1990 and 2023, encompassing 619 studies, colistin resistance was reported to rise from 2% during the 2006–2010 period to 5% in the 2020–2023 period, with the estimated overall prevalence of colistin resistance reported as 1% [49]. In a meta-analysis, the highest resistance rates for pseudomonal infections were observed in Egypt (15%) and Pakistan (13%) [55]. Another noteworthy finding was that *P. aeruginosa* isolates recovered from respiratory specimens of patients with cystic fibrosis exhibited the highest levels of colistin resistance, reaching rates of 5–7% [55,66] (see Table 2).

### 5.6. Colistin Resistance Among A. baumannii

A prospective international study conducted in 2019, which analyzed 177 cases of ventilator-associated pneumonia reported a colistin resistance rate of 1.1% [67]. In a meta-analysis assessing 398 studies published between 2000 and 2023, the prevalence of colistin resistance among *A. baumannii* isolates was 4% (95% CI: 3–5%), with South America identified as the region exhibiting the highest resistance rate [53]. Likewise, a separate review of 167 studies conducted between 2001 and 2017 found that colistin resistance among *Acinetobacter* strains ranged from 2% to 5%, depending on the antimicrobial susceptibility testing method employed [54]. In another analysis focusing on *Acinetobacter* strains, colistin resistance rates were reported as 7% in Western Europe, 6% in South America, and 4% in Asia. Specifically, resistance rates of 2%, 3%, and 6% were reported from China, Türkiye, and Iran, respectively. A recent meta-analysis including 734 studies reported remarkably elevated resistance rates in several countries including Israel (59%), France and the United Arab Emirates (50% each), Argentina (46%), and Greece (18%), highlighting serious and growing concerns [53]. On the other hand, analyses of resistance rates stratified by year of publication in several studies have shown an increase in colistin resistance during the period from 2019 to 2020, coinciding with the COVID-19 pandemic. However, a subsequent decline in resistance levels was observed as the pandemic came under control. This pattern has been attributed to the widespread use of antibiotics during the COVID-19 pandemic [50,53,55]. (see Table 2).

## 6. Colistin Based Antimicrobial Treatment Strategies

### 6.1. Impact of Multi Drug Resistance on Treatment

Infections caused by GNB tend to be severe and, owing to their tendency for multidrug resistance, multiple dissemination pathways, and ability to exhibit concurrent resistance to numerous antibiotic classes, they are regarded as one of the most significant global health threats [1,18]. Added to that, MDR-GNB further complicate the outcome of the patients [1,8,15]. Therefore, the World Health Organization first published the list of priority pathogens requiring urgent development of new antibiotics in 2017. In the most recent update released in 2024, the list includes carbapenem-resistant Enterobacteriaceae (CRE), as well as carbapenem-resistant *Pseudomonas aeruginosa* and *Acinetobacter baumannii* [68,69]. In its most recent guidelines on the management of GNB infections, the Infectious Diseases Society of America (IDSA) emphasizes that novel β-lactam/β-lactamase inhibitor combinations including ceftazidime–avibactam, meropenem–vaborbactam, imipenem–cilastatin–relebactam, ceftolozane–tazobactam, and cefiderocol should be reserved primarily for infections caused by carbapenem-resistant organisms, and that identification of the specific carbapenem resistance mechanism is essential for optimal antibiotic selection [1,2]. However, until access to newer antimicrobial agents becomes more widespread, the treatment of MDR-GNB infections often necessitates the use of older and, in some cases, more toxic agents like colistin, trimethoprim–sulfamethoxazole, fluoroquinolones, or nitrofurantoin [70].

### 6.2. Guideline-Based Use of Colistin for Carbapenem Resistant Bacteria

Colistin has traditionally been used as a last-resort option in combination regimens for the treatment of MDR-GNB infections; however, colistin resistance has now emerged as a major clinical concern. In regions where CRE are endemic, the coexistence of carbapenem and colistin resistance has been increasingly reported [44]. Colistin-resistant infections are recognized to result in higher treatment failures, longer hospital stays, and increased morbidity and mortality, particularly among critically ill patients [63,71,72].

Use of colistin as monotherapy is considered appropriate by the IDSA only for the treatment of uncomplicated cystitis caused by CRE [2]. Similarly, the European Society of Clinical Microbiology and Infectious Diseases (ESCMID) guideline recommend polymyxins alone for mild or low-risk CRE infections when in vitro susceptibility is documented, but do not consider them appropriate for severe infections, including CRE-associated bloodstream infections and ventilator-associated pneumonia due to limited efficacy and significant toxicity [73].

Both IDSA and ESCMID guidelines advise against combination therapy with polymyxins and other agents for CRE infections when newer β-lactam options are available. In settings where these agents are not accessible, a combination of a polymyxin with tigecycline or eravacycline may be considered for bacteremic cases of skin and soft tissue infections or complicated intra-abdominal infections. Nevertheless, the clinical role of combinations such as colistin-meropenem, colistin-aminoglycoside, or colistin-tigecycline remains uncertain [2,73].

### 6.3. Antimicrobial Combination Strategies

Combination therapy remains a cornerstone in the management of colistin-resistant GNB infections [9,10,61,62]. Colistin-based combinations have demonstrated synergistic activity with a wide range of antimicrobial agents, including rifampin, azithromycin, fusidic acid, linezolid, vancomycin, aztreonam, ceftazidime, imipenem, and tigecycline, against colistin-resistant GNB [74,75,76]. In patients with carbapenem-resistant *K. pneumoniae* infections, combining tigecycline with colistin has been reported to reduce the rate of colistin resistance development during treatment [77].

Antimicrobial treatment options for different infection sites caused by carbapenem-resistant enteric pathogens, stratified by carbapenemase type. For metallo-β-lactamase-producing strains, therapy relies mainly on ceftazidime/avibactam combined with aztreonam or on cefiderocol, with additional agents such as eravacycline or plazomicin depending on the infection site. For non-metallo-β-lactamase carbapenemase-producing strains with a meropenem MIC < 16 mg/L, treatment options include ceftazidime/avibactam-based regimens or high-dose, prolonged-infusion meropenem in combination with other agents (e.g., fosfomycin, gentamicin, or tigecycline), tailored to the site of infection. Overall, infection site-specific and resistance mechanism-guided combination therapy should be mandated [78,79]. Potential combination strategies are presented in Table 3 (adapted from [78,79]). In preclinical models, colistin combined with clarithromycin was effective at clinically relevant exposures in a murine thigh infection model involving MCR-1–producing, colistin-resistant *K. pneumoniae* [80].

In vitro synergy studies further support the utility of selected colistin-based regimens. In an analysis of colistin-antibiotic interactions against colistin and carbapenem-resistant *K. pneumoniae* isolates, strong synergy (fractional inhibitory concentration index [FICI] ≤ 0.5) was observed with clindamycin, erythromycin, minocycline, levofloxacin, chloramphenicol, and rifampin, whereas imipenem, meropenem, and amikacin demonstrated only weak or no synergy (FICI > 0.5). The mean duration of antecedent antibiotic exposure in this cohort was 20 days [81]. Colistin combination strategies have been particularly well studied in *A. baumannii* infections. Several agents including econazole, tigecycline, meropenem, rifampin, fosfomycin, amikacin, tobramycin, ampicillin/sulbactam, minocycline, eravacycline, ceftazidime–avibactam, trimethoprim–sulfamethoxazole, and azithromycin have been identified as potentially effective partners with colistin for the treatment of colistin-resistant *A. baumannii* [32]. Notably, combinations of carbapenems with antibiotics that are traditionally inactive against Gram-negative organisms, such as rifampin, glycopeptides, daptomycin, and fusidic acid, have demonstrated synergistic activity against *A. baumannii* [82]. The efficacy of these unconventional combinations is thought to be mediated by polymyxin-induced disruption of the outer membrane, resulting in increased permeability and enhanced intracellular penetration of otherwise excluded antimicrobial agents. Consistent with this mechanism, colistin in combination with fosfomycin or fusidic acid has been associated with improved microbiological and clinical outcomes, and fusidic acid has been proposed to reduce the emergence of colistin resistance [83].

Collectively, current data indicate that colistin-based combination regimens represent a biologically possible and clinically relevant approach for managing colistin-resistant GNB infections, especially when therapeutic options are severely limited.

### 6.4. Use of Inhaler Colistin

In a randomized study of 149 patients with Gram-negative ventilator-associated pneumonia, inhaled colistin administered in combination with intravenous colistin and imipenem led to faster resolution of respiratory failure and more rapid microbial eradication compared with intravenous colistin and imipenem alone; however, clinical cure rates, length of intensive care unit stay, and mortality were comparable between the two groups [84]. In another randomized trial involving 100 patients with ventilator-associated pneumonia caused by *A. baumannii* and *P. aeruginosa*, adjunctive inhaled colistin was compared with inhaled saline in addition to intravenous antibiotics. Although inhaled colistin did not improve clinical outcomes and was associated with a higher rate of bronchospasm, it increased microbial eradication from respiratory secretions [85]. In contrast, a meta-analysis of nine observational studies found that the addition of nebulized colistin to intravenous colistin for the treatment of MDR hospital-acquired pneumonia was associated with higher clinical cure rates and lower mortality compared with intravenous therapy alone [86]. Overall, current evidence suggests that while adjunctive inhaled colistin may enhance microbiological eradication in pulmonary infections, the cornerstone of anti-infective therapy, its impact on clinical outcomes remains inconsistent and warrants further well-designed randomized studies.

## 7. Non-Antibiotic Therapies

### 7.1. Adjuvants

Adjuvants are non-antibiotic compounds that enhance the activity of antibiotics such as colistin. These molecules increase the efficacy of antibiotics by both facilitating their penetration into bacterial cells and inhibiting their efflux, thereby restoring bacterial susceptibility to the antibiotic. Kaempferol, 2-aminoimidazole, chrysin, curcumin, AOA-2, panduratin A, IMD-0354, NMD-27, auranofin, essential oils of clove and thyme, disulfiram, and capsaicin have been reported as effective adjuvants when used in combination with colistin against colistin-resistant *A. baumannii* strains [32].

### 7.2. Drug Repurposing

Drug repurposing refers to the utilization of the known pharmacological properties of existing drugs to treat intrinsically resistant infections. Compared with the development of new drugs, this approach is faster and more cost-effective. Antihelmintic agents such as niclosamide, closantel, and oxyclozanide; antifungal agents such as tavaborole and econazole; and anticancer drugs including mitomycin C, 5-fluorouracil, and tamoxifen have been shown to restore colistin activity when used in combination with colistin [32].

### 7.3. Antimicrobial Peptides

Antimicrobial peptides are polypeptides composed of various amino acids with differing molecular weights and are found in nearly all living organisms, ranging from simple prokaryotes to complex eukaryotes. They play important roles in the direct killing of viruses, bacteria, fungi, and parasites, as well as in the modulation of immune processes such as activation of immune cells, wound healing, angiogenesis, and inflammation [87]. Numerous studies on antimicrobial peptides have demonstrated that they are effective against MDR, XDR, and colistin-resistant *A. baumannii*, either when used alone or in combination with colistin. Compared with conventional empirical antibiotics, antimicrobial peptides exhibit a lower tendency for resistance development, rapid bactericidal activity, and broad-spectrum antimicrobial effects [87,88]. Peptides such as Esc (1–21), melittin, indolicidin, mastoparan, Ω76, NuriPep 1653, Cec4, 2K4L, LS-sarcotoxin, and LS-stomoxin have been shown to be effective against *A. baumannii* [32].

### 7.4. PYED-1 and Colistin Synergy

The steroid-structured compound, pregnadiene-11-hydroxy-16,17-epoxy-3,20-dione-1 (PYED-1), which exhibits antibiofilm, antimicrobial, and antivirulence properties against Gram-positive and GNB as well as Candida species, has demonstrated synergistically enhanced antimicrobial activity against colistin-resistant *A. baumannii* strains when combined with colistin [89]. The results seem promising.

### 7.5. Bacteriophage Therapy

Bacteriophages, viruses that infect bacteria, have been intensively investigated for use in various therapeutic applications for many years. Accordingly, increasing attention over the past two decades has focused on the antibacterial potential of phage-encoded lysins. The biochemical specificity of lysins is generally broader than that of intact bacteriophages. Lysins include peptidoglycan hydrolases and endolysins that degrade the bacterial peptidoglycan layer [90]. When specific lysins are applied exogenously to Gram-positive bacteria, they damage the peptidoglycan layer, leading to rapid osmotic lysis. However, this effect is largely reduced in GNB due to the protective outer membrane that limits access to the peptidoglycan. Nevertheless, some lysins exhibit intrinsic antibacterial activity against GNB when applied externally. *A. baumannii* appears to be susceptible to this group of lysins with natural antibacterial activity in particular. For example, combining lysins with EDTA or weak acids (such as citric or malic acid) acts as an outer membrane destabilizer [32,90]. Phage therapy has emerged as one of the most promising therapeutic approaches gaining momentum for the management of colistin-resistant *A. baumannii* infections. A lysin derived from bacteriophage PMK34, when used in combination with colistin, resulted in up to a 32-fold reduction in the MIC of colistin and restored susceptibility in colistin-resistant strains [90]. Additionally, the phage effectively reduced biofilms and prevented the formation of new ones [32,90]. In a clinical case, a MDR *A. baumannii* infection unresponsive to antibiotics including colistin and tigecycline was treated with nine bacteriophages targeting the pathogen, administered intravenously and locally into abscesses. Minocycline was added to this regimen on the fifth day, and the combined therapy successfully eradicated the infection [91].

### 7.6. Fecal Microbiota Transplantation

Fecal Microbiota Transplantation (FMT) is a therapeutic approach aimed at restoring intestinal microbial diversity and suppressing pathogenic microorganisms by transferring fecal material from a healthy donor to an individual with gastrointestinal colonization. Its efficacy has been well established in recurrent *Clostridioides difficile* infections, and it has also shown promising results in the decolonization of multidrug-resistant organisms including carbapenem-resistant *K. pneumoniae*, thereby preventing subsequent systemic infections [92,93]. Although data on mcr-positive bacteria remain limited, some case series have reported partial eradication of intestinal carriage of mcr-1–positive *E. coli* following repeated FMT procedures. However, the lack of standardization regarding donor selection and screening, potential risks in immunosuppressed patients, uncertainty about the long-term durability of decolonization, and variability in administration routes (oral capsules, colonoscopy, or nasogastric delivery) pose significant challenges to the widespread implementation of FMT [94].

### 7.7. Nanoparticles

Nanotechnology represents one of the most promising approaches in the fight against antimicrobial resistance by offering novel therapeutic options and thereby helping to preserve existing antibiotic resources. Although the antibacterial mechanisms of nanoparticles are not yet fully understood, their small particle size and charged surfaces enable them to readily penetrate pathogenic cells. They can damage bacterial membranes, generate reactive oxygen species, and inhibit DNA synthesis, ultimately leading to programmed cell death [95,96]. Studies have shown that combining membrane-disrupting antibiotics such as colistin with silver nanoparticles enhances their antibacterial activity. Nanoparticles can disrupt the outer membrane and cell wall, thereby potentiating colistin activity and facilitating the antibiotic’s passage to the cytoplasmic membrane and its target site [96]. In addition, silver nanoparticles at specific concentrations have been shown not only to exert intrinsic antimicrobial effects but also to downregulate the expression of genes associated with virulence and biofilm formation [97].

### 7.8. Antimicrobial Photodynamic Therapy

Traditionally, phototherapy has been used since ancient times in Greece, Egypt, and India for the treatment of skin diseases. Photodynamic therapy was first introduced as an antimicrobial treatment in the early 1990s, when it was applied locally to manage antibiotic-resistant infections. Regardless of antimicrobial resistance profiles, photodynamic therapy has been used as a local therapy in combination with systemic treatments, and to date, the development of resistance has been rarely reported [98]. Experimental studies have demonstrated that when photodynamic therapy is used in combination with colistin, a strong synergistic effect against *A. baumannii* is observed. Specifically, this combination resulted in more than an 11-fold reduction in the MIC of colistin against colistin-resistant *A. baumannii*, along with an 83.7% reduction in bacterial counts [99,100].

### 7.9. CRISPR Technology

The CRISPR–Cas system is an intrinsic adaptive immune defense mechanism acquired by bacteria, protecting genomic stability by resisting the invasion of exogenous genetic elements such as plasmids and bacteriophages. In addition to being a key component of the prokaryotic immune system, CRISPR–Cas systems have also been implicated in modulating bacterial virulence and counteracting the spread of antibiotic resistance [101]. Based on the composition of Cas proteins, CRISPR–Cas systems are classified into two main classes, six types (I–VI), and more than 30 subtypes. Approximately 39% of bacterial genomes harbor CRISPR–Cas systems, while nearly 70% of pathogenic bacteria possess Type I CRISPR–Cas systems [101]. Type I-F is the most prevalent CRISPR–Cas system in *A. baumannii*. Recent studies have shown that *A. baumannii* utilizes the Type I-Fb CRISPR–Cas system to limit the dissemination of antibiotic resistance genes [102]. In a study by Wang et al., CRISPR/Cas9 was used to eliminate plasmids from *E. coli* isolates, rendering the bacteria more susceptible to antibiotics [103].

## 8. Conclusions

The reintroduced reliance on colistin as a last-line therapy for multidrug-resistant Gram-negative infections has exposed a critical vulnerability in current antimicrobial practices. While colistin remains essential, the rapid global expansion of resistance driven by chromosomal mutations, transferable mcr genes, and selective pressures across human, animal, and environmental reservoirs threatens its long-term viability. The frequent co-occurrence of colistin resistance with carbapenemase genes on mobile genetic elements highlights the fragility of remaining therapeutic options and underscores the necessity of a coordinated One Health approach.

A major future priority lies in advancing mechanistic understanding of resistance evolution under clinical and ecological conditions. Key unresolved questions include the fitness costs and stability of resistance determinants, the potential for resistance reversal, and the role of non-clinical reservoirs in sustaining transmission. Addressing these gaps is essential for informing both therapeutic strategies and surveillance efforts.

Clinically, colistin’s narrow therapeutic window, nephrotoxicity, and complex pharmacokinetics continue to complicate its use. Although optimized dosing strategies and improved susceptibility testing have improved safety, evidence supporting colistin-based combination therapy remains largely derived from in vitro studies and observational data. Well-designed randomized trials are urgently needed to define the clinical contexts in which mechanism-guided, site-specific combination regimens provide meaningful benefit, particularly in settings with limited access to newer antimicrobials.

Innovative non-antibiotic approaches including antimicrobial peptides, resistance-modifying adjuvants, bacteriophage and lysin therapy, nanoparticles, and CRISPR-based technologies offer promising alternatives to restore colistin activity or bypass conventional resistance pathways. However, substantial translational barriers persist, including challenges related to clinical implementation, regulatory approval, safety, and clinical validation.

Finally, scientific advances must be complemented by robust antimicrobial stewardship, integration of stewardship principles into postgraduate training [104], reduced agricultural use, and strengthened infection prevention and control. Preserving colistin will ultimately require coordinated global action; without it, resistance to last-resort agents risks accelerating the transition toward a post-antibiotic era.

## Figures and Tables

**Table 1:** The features of MCR genes associated with plasmid-mediated colistin resistance.

Gene	First Report	Hosts	Plasmid Location	Colistin MIC	
mcr-1	2015, China, pig	Enterobacterales	IncI2, IncX4, IncHI2	2–8 mg/L	Most globally prevalent
mcr-2	2016, Belgium, pig	*E. coli*	IncX4	2–8 mg/L 4–8 mg/L	Limited global spread
mcr-3	2017, China, pig	*E. coli*, *Salmonella*, *Aeromonas*	IncHI2, IncP, IncFII	4–8 mg/L ≥4–8 mg/L	Environmental reservoirs
mcr-4	2017, Italy, swine	*Salmonella enterica*, *E. coli*	IncHI2, CoIE-like	4–8 mg/L	Sporadic
mcr-5	2017, Germany, animal feces, human	*Salmonella*, *E. coli*	IncX1, chromosomal	8 mg/L 4 mg/L	Food chain
mcr-6	2017, UK, pig	*Moraxella* spp.	chromosomal	1–2 mg/L	Rare
mcr-7	2018, China, chicken	*K. pneumoniae*	IncI1	4–8 mg/L	Limited distribution
mcr-8	2018, China, cattle, human	*K. pneumoniae*, *Raoultella*	IncFII(K), IncHI2	8–16 mg/L 16 mg/L	Clinical outbreaks in Asia
mcr-9	2019, USA, human, animal, food, environment	*Serratia*, *Morganella*	IncHI2, IncFII	≤2 mg/L	Widespread
mcr-10	2020, China, clinical	*Enterobacter* spp., *Klebsiella*	IncFII(K)	4 mg/L 4 -> 16–128 mg/L	Emerging

**Table 2:** Global and Regional Resistance Colistin Rates of Major Gram-Negative Pathogens.

Microorganism	Reference	Global Resistance Rate (%)	Region/Country	Resistance Rate (%)
*K. pneumoniae*	[49]	13	Asia	30–50
			India	40–60
			China	30–45
			Indonesia	25–40
			Pakistan	30
			Europe (overall)	10–20
			Italy	10–20
			Greece	10–15
			Americas (overall)	5–10
			Brazil	15–25
			Türkiye	20–30
	[48]		Africa (overall)	21.6
			East Africa	42.3
			West Africa	37.1
			Southern Africa	17.1
			North Africa	13.0
	[50]		Türkiye (national data)	13.4
Enterobacteriaceae	[51]	9.1	Africa (overall)	26.7
	[52]		Southern Africa	50.9
			Algeria	49.9
			Tanzania	36.1
			Egypt	19.9
*A. baumannii*	[53]	4	Asia (overall)	4
	[54]		China	12
	[53]		Western Europe	7
			Eastern Europe	1
			France (local data)	50
			Greece (local data)	18
			Italy	2
			Americas (overall)	5
			Brazil	8
			Argentina (single-center)	46
			Middle East–Israel (single-center)	59
			Iraq	19
			Lebanon	17.5
*P. aeruginosa*	[55]	1	Africa (overall)	4
			Egypt	15
			Pakistan	13
			Cystic fibrosis patients	7

**Table 3:** Possible antibiotic treatment options in colistin and carbapenem-resistant infections according to meropenem MIC values.

Infection Site	MBL-Producing Strains	Non–MBL Carbapenemase—Producing Strains	
		Meropenem MIC ≤ 16 mg/L	Meropenem MIC > 16 mg/L
Bloodstream Infection	CAZ-AVI + aztreonam Cefiderocol	CAZ-AVI High-dose prolonged-infusion meropenem + fosfomycin High-dose prolonged-infusion meropenem + gentamicin High-dose prolonged-infusion meropenem + fosfomycin + gentamicin	CAZ-AVI CAZ-AVI ± fosfomycin or gentamicin Consider fosfomycin plus gentamicin in case of resistance to CAZ-AVI Cefiderocol Plazomicin MER-VAB (inactive against OXA-48 producers)
Hospital-Acquired Pneumonia (including VAP)	CAZ-AVI + aztreonam Cefiderocol Eravacycline	High-dose prolonged-infusion meropenem + fosfomycin CAZ-AVI + fosfomycin + gentamicin	CAZ-AVI + fosfomycin ± gentamicin Consider fosfomycin plus gentamicin in case of resistance to CAZ-AVI MER-VAB (inactive against OXA-48 producers)
Intra-abdominal Infection	CAZ-AVI + aztreonam Cefiderocol	CAZ-AVI + tigecycline + gentamicin High-dose prolonged-infusion meropenem + tigecycline + gentamicin	CAZ-AVI + tigecycline ± gentamicin CAZ-AVI + tigecycline ± fosfomycin Plazomicin MER-VAB (inactive against OXA-48 producers)
Urinary Tract Infection	CAZ-AVI + aztreonam Cefiderocol Plazomicin	CAZ-AVI + fosfomycin + gentamicin High-dose prolonged-infusion meropenem + fosfomycin + gentamicin Fosfomycin trometamol (uncomplicated UTI)	CAZ-AVI ± fosfomycin ± gentamicin Consider fosfomycin + gentamicin in case of resistance to CAZ-AVI MER-VAB (inactive against OXA-48 producers)
Complicated Skin and Soft Tissue Infection	CAZ-AVI + aztreonam Cefiderocol	High-dose prolonged-infusion meropenem + tigecycline CAZ-AVI + tigecycline	CAZ-AVI ± tigecycline CAZ-AVI ± fosfomycin CAZ-AVI + tigecycline ± fosfomycin

MBL: Metallo-β-lactamase, CAZ-AVI: Ceftazidime/avibactam, MER-VAB: Meropenem/Vaborbactam.

## Data Availability

No new data were created or analyzed in this study. Data sharing is not applicable to this article.
